# Development of large-scale mosquito densovirus production by *in vivo* methods

**DOI:** 10.1186/s13071-019-3509-5

**Published:** 2019-05-22

**Authors:** Yan Sun, Yunqiao Dong, Jing Li, Zetian Lai, Yanqiang Hao, Peiwen Liu, Xiaoguang Chen, Jinbao Gu

**Affiliations:** 1 Guangdong Provincial Key Laboratory of Tropical Disease Research, Department of Pathogen Biology, School of Public Health, Southern Medical University, Guangzhou, 510515 Guangdong China; 2 Reproductive Medical Centre of Guangdong Women and Children Hospital, Guangzhou, 511442 Guangdong China

**Keywords:** Mosquito-borne diseases, Vector control, Mosquito densovirus, Densovirus production, Bioinsecticides

## Abstract

**Background:**

Mosquito-borne diseases (MBDs) cause a significant proportion of the global infectious disease burden. Vector control remains the primary strategy available to reduce the transmission of MBDs. However, long-term, wide-scale and large-scale traditional chemical pesticide application has caused significant and increased negative effects on ecosystems and broader emerging insecticide resistance in vectors; therefore, the development of a novel alternative approach is urgently needed. Mosquito densoviruses (MDVs) are entomopathogenic viruses that exhibit a narrow host range and multiple transmission patterns, making MDVs a great potential bioinsecticide. However, the application process has been relatively stagnant over the past three decades. The major obstacle has been that viruses must be produced in mosquito cell lines; therefore, the production process is both expensive and time-consuming.

**Methods:**

In our study, two wild-type (wt) MDVs, AaeDV and AalDV-3, and a recombinant rAaeDV-210 were used to infect the Aag2 and C6/36 mosquito cell lines and the 1st–2nd-instar and 3rd–4th-instar larvae of *Ae. albopictus*, *Ae. aegypti* and *Cx. quinquefasciatus*. Viral titers and yields in cells, media, larvae and rearing water and total viral yield were evaluated. Three kinds of virus displayed higher maximum virus titers *in vivo* than *in vitro*, and they displayed higher maximum viral yields in rearing water.

**Results:**

The three viruses displayed higher total maximum viral yields in C6/36 cells than in Aag2 cells. The three viruses displayed higher total maximum viral yields in *Aedes* mosquitoes than in *Culex* mosquitoes. Higher viral yields were produced by 1st–2nd-instar larvae compared to 3rd–4th-instar larvae. The recombinant viruses did not display significantly lower yields than wt viruses in nearly all samples. In summary, by using 100 1st–2nd-instar *Aedes* mosquito larvae in 200 ml of rearing water, more than 10^13^ genome equivalents (geq) MDV yield can be obtained.

**Conclusions:**

Considering the lower production cost, this *in vivo* method has great potential for the large-scale production of MDVs. MDVs exhibit promising prospects and great potential for mosquito control in the future.

**Electronic supplementary material:**

The online version of this article (10.1186/s13071-019-3509-5) contains supplementary material, which is available to authorized users.

## Background

Mosquito-borne diseases (MBDs), such as malaria, dengue fever, yellow fever, chikungunya, Zika, hemorrhagic fevers and lymphatic filariasis, cause a significant fraction of the global infectious disease burden; indeed, nearly 700 million people worldwide are infected with MBDs each year, resulting in over one million deaths [1]. Vector control remains the primary strategy available to reduce the transmission of MBDs. Synthetic chemical insecticides have been traditionally used as a standard method for vector control and play a key role in public health as part of integrated mosquito management (IMM) programs for disease control and prevention and dramatically reduce the risk of MBDs, particularly in the case of malaria [2]. However, long-term, wide-scale and large-scale traditional chemical pesticide application has certainly shown significant and increased negative effects on ecosystems [3]. Additionally, the broadly emerging insecticide resistance of vectors poses a major threat to effective prevention and control of MBDs. In view of these pending issues, the development of novel alternative approaches that can be applied to mosquito control is urgently needed. Biological control of vectors using insect specific pathogens or symbiotic microorganisms, such as fungi, bacteria or viruses, can reduce vector populations or vectoral capacity, and hence disease transmission, and be produced as eco-friendly, highly effective, specific, economic, biodegradable bioinsecticides [4].

Entomopathogenic viruses have been identified in more than one thousand species from at least 13 different insect orders [5]. These insect pathogenic viruses can be divided into two categories: those that produce occlusion bodies in the insect host cells are occluded viruses (OVs), whereas those that are not occluded in protein bodies are no-occluded viruses (NOVs) [6]. The most common OVs of mosquitoes are the nuclear polyhedrosis viruses (NPVs) (family *Baculoviridae*), such as *Culex nigripalpus* nuclear polyhedrosis virus (CuniNPV) and cytoplasmic polyhedrosis viruses (CPVs) (family *Reoviridae*, genus *Cypovirus*) [7, 8]. The main NIVs in mosquitoes are mosquito iridoviruses (MIVs) (family *Iridoviridae*, genus *Chloriridovirus*) and densoviruses (DVs) [9].

Mosquito densoviruses (MDVs) are currently included in the genus *Brevidensovirus* of the subfamily *Densovirinae* in the family *Parvoviridae*. The virion is a small icosahedral, non-enveloped DNA virus with a diameter of 20 nm. The viral genome is a linear, single-stranded DNA (ssDNA) molecule approximately four kilobases long. MDVs exhibit strong host specificity that is restricted to members of the family Culicidae in the order Diptera. MDVs can invade and proliferate in multiple organs and tissues of mosquitoes, and infection with MDVs can cause larval death or deformation [10]. Moreover, MDVs extend the larval stage and decrease the lifespan and body size of adults [11]. Furthermore, their pathogenicity can be significantly improved by genetic engineering techniques, such as altering the vector used to express the insect-specific toxin, gene or specific shRNA or artificial miRNA that targets genes essential for the development, growth or physiology of mosquitoes [12–14]. MDVs can be transmitted in mosquito populations through horizontal transmission in larval habitats and/or by vertical transmission. Thus, these biological and pathogenic characteristics confer the potential for MDVs as biological control agents [11]. Considering that the Biokiller Cockroach Killer Bait Gel, the first densovirus biopesticide product, which mainly contains *Periplaneta fuliginosa* densonucleosis virus (PfDNV, genus *Ambidensovirus*, family *Parvoviridae*), was commercialized in China [15], the use of MDVs and/or recombinant MDV biopesticides is within reach.

Despite promising application prospects, the standardization of the large-scale production of MDVs remains a major barrier for the progression from laboratory to field. In this study, we analyzed the accumulation of wild-type and recombinant MDVs in mosquito cell lines, including the *Ae. albopictus* C6/36 cell lines and *Ae. aegypti* Aag2 cell lines, as well as in three different species of mosquito larvae, *Ae. aegypti*, *Ae. albopictus* and *Cx. quinquefasciatus*. The maximum virus titer and viral yield were evaluated and compared. Finally, we developed a novel and more economical method for the mass production of MDVs in mosquito larvae, which will provide sufficient amounts of virus for bioassays, future moderate-scale field testing and even large-scale field application.

## Methods

### Mosquito cell transfection and virus production

pUCA and pUCP were the infectious clones of AaeDV (GenBank: M37899) and AalDV-3 (or APeDNV, GenBank: AY310877) that contained the full genomic DNA of AaeDV (3981 nt) and AalDV-3 (4006 nt), respectively, and were kindly provided by Professor Erica Suchman and Jonathan Carlson (Colorado State University, Fort Collins, CO, USA). pUCA-210 is an infectious clone of non-defective recombinant densovirus vectors that can express endogenous miRNA-210 in *Ae. albopictus*. The construction of plasmids has been previously described in detail [16, 17].

Wild-type viruses AaeDV and AalDV-3 and recombinant virus rAaeDV-210 were generated by transfecting the corresponding infectious clones pUCA, pUCP and pUCA-210 into C6/36 cell lines according to the manufacturer’s protocol. Before transfection, cells were plated at a density of 2.5 × 10^6^ cells per 25 cm^2^ flask. The cells were incubated at 28 °C until the cells were 80 to 90% confluent. The transfection of plasmids was performed using Lipofectamine 2000 (Thermo Fisher Scientific, Waltham, MA, USA), according to the manufacturer’s protocol. Supercoiled plasmids used for transfection were prepared using a GeneJET Endo-Free Plasmid MaxiPrep Kit (Thermo Fisher Scientific). Cells were typically harvested 5 days post-transfection using cell scrapers, lysed by repeat freezing-thawing treatment, and then centrifuged for 10 min at 1000×*g*. The supernatants were kept as viral stocks.

### Viral accumulation in mosquito cells

To explore the proliferation capability of AaeDV, AalDV-3 and recombinant virus rAaeDV-210 in mosquito cells, C6/36 and Aag2 cells were seeded in 24-well plates (C6/36 cells: 1 × 10^5^ cells/well, Aag2 cells: 1 × 10^6^ cells/well) and incubated with 1 ml of media containing virus at a final concentration of 3.00 × 10^9^ genome equivalents per ml (geq/ml) at 28 °C. After incubation for 24 h, the medium was removed, and the cells were washed 3 times with fresh RPMI-1640 medium (Schneider’s *Drosophila* medium for Aag2 cells) to remove residual virus. Then, 1 ml of fresh medium was added, and the cells were returned to the incubator for regular culture.

Viral titers in the cell culture supernatant and in the cells themselves were determined by quantitative real-time PCR (qPCR). First, to determine the titer of the extracellular viruses, samples from the cell culture were collected at serial time points post-infection [0, 2, 4, 6, 8 and 10 days post-infection (dpi)] and then centrifuged for 10 min at 1000× *g*. Supernatants were used to extract viral genomic DNA using a Viral DNA Kit (Omega Biotek, Norcross, GA, USA). To determine the titer of the intracellular virus, the supernatants were removed, and the infected cells were washed 3 times with 1× PBS. The cells were collected at serial time points post-infection (0, 2, 4, 6, 8 and 10 dpi); the genomic DNA was then isolated for qPCR.

### Larval infection and densovirus detection

To explore the percentage of infected mosquito larvae when exposed to MDV, the 1st–2nd-instar and 3rd–4th-instar larvae (*n = *100 per group) of *Ae. albopictus*, *Ae. aegypti* and *Cx. quinquefasciatus* larvae were infected with AaeDV, AalDV-3 and rAaeDV-210 by exposure to 1 ml of sterile water in a beaker containing virus with a final concentration of 2.00  × 10^10^ geq/ml. After incubation for 24 h at 28  °C, the larvae were washed three times with deionized water and then transferred to pans containing 200 ml sterile water. After 1 dpi, larvae were washed three times with deionized water again, and the DNA was isolated using a TIANcombi DNA Lyse&Det PCR Kit (Tiangen Biotech, Beijing, China). PCR amplification was then performed using Maxima Hot Start Green PCR Master Mix (Thermo Fisher Scientific) with gene-specific primers. The final washed water was used as a control. *Ae. albopictus* ribosomal protein 7 gene (AalRpS7, GenBank: JN132168), *Ae. aegypti* RpS7 (AaeRpS7, VectorBase accession number: AAEL009496) and *Cx. quinquefasciatus* RpL8 (CquRpL8, GenBank: XM_001841875) were used as controls, respectively.

### Viral accumulation in mosquito larvae

The 1st–2nd-instar and 3rd–4th-instar *Ae. albopictus*, *Ae. aegypti* and *Cx. quinquefasciatus* larvae (*n = *100 per group) were exposed to AaeDV, AalDV-3 and recombinant virus rAaeDV-210 at a concentration of 2.00 × 10^10^ geq/ml in 1 ml of sterile water. The blank control group was exposed to virus-free C6/36 cell culture medium under conditions identical to the treatment groups. After incubation for 24 h at 28 °C, the larvae, along with water, were transferred back to the pans, and then sterile water was added until the viral concentration was 1.00 × 10^8^ geq/ml; larvae were fed regularly. The 1st–2nd-instar larval rearing water was collected at serial time points post-infection (1, 3, 5, 7, 9 and 11 dpi), whereas the 3rd–4th-instar larval rearing water was collected at 1, 3, 5 and 7 dpi. The virus in the rearing water was titered by qPCR. The larvae were also collected at the same time point, and the virus *in vivo* was also titrated by qPCR.

### Quantitative real-time PCR

Non-encapsidated genomic DNA was removed by treatment with TURBO DNase (Ambion, Austin, TX, USA) at 37 °C for 1 h. The total encapsidated genomic DNA was extracted using a Viral DNA Kit (Omega Biotek). The virus genome copy numbers in cell culture media, cells, rearing water and larvae *in vivo* were determined by a SYBR green-based real-time quantitative PCR assay with FastFire qPCR PreMix (Tiangen Biotech). A series of known concentrations of linear plasmids with pUCA were used to construct a standard curve. The set of primers used annealed to the conserved region of the viral NS1 gene. These procedures were performed essentially as described previously [18]. The sequences of primers used in the PCR and qPCR are shown in Additional file 1: Table S1.

### Statistical analysis

Comparison of the percentage of infected larvae between groups was performed using a Chi-square test. Virus titer and viral yield in cells, cell culture media, larvae and rearing water and total viral yield at different times and in different groups were compared using one-way analysis of variance (ANOVA) followed by Fisher’s least significant difference test (LSD). Student’s t-test was performed to determine differences between two groups. The *P*-value for statistical significance is defined as *P* < 0.05. SPSS software v.19.0 (SPSS Inc. Chicago, IL, USA) was used for data analysis.

## Results

### The accumulation of the MDVs in mosquito cells

**Fig. 1:**
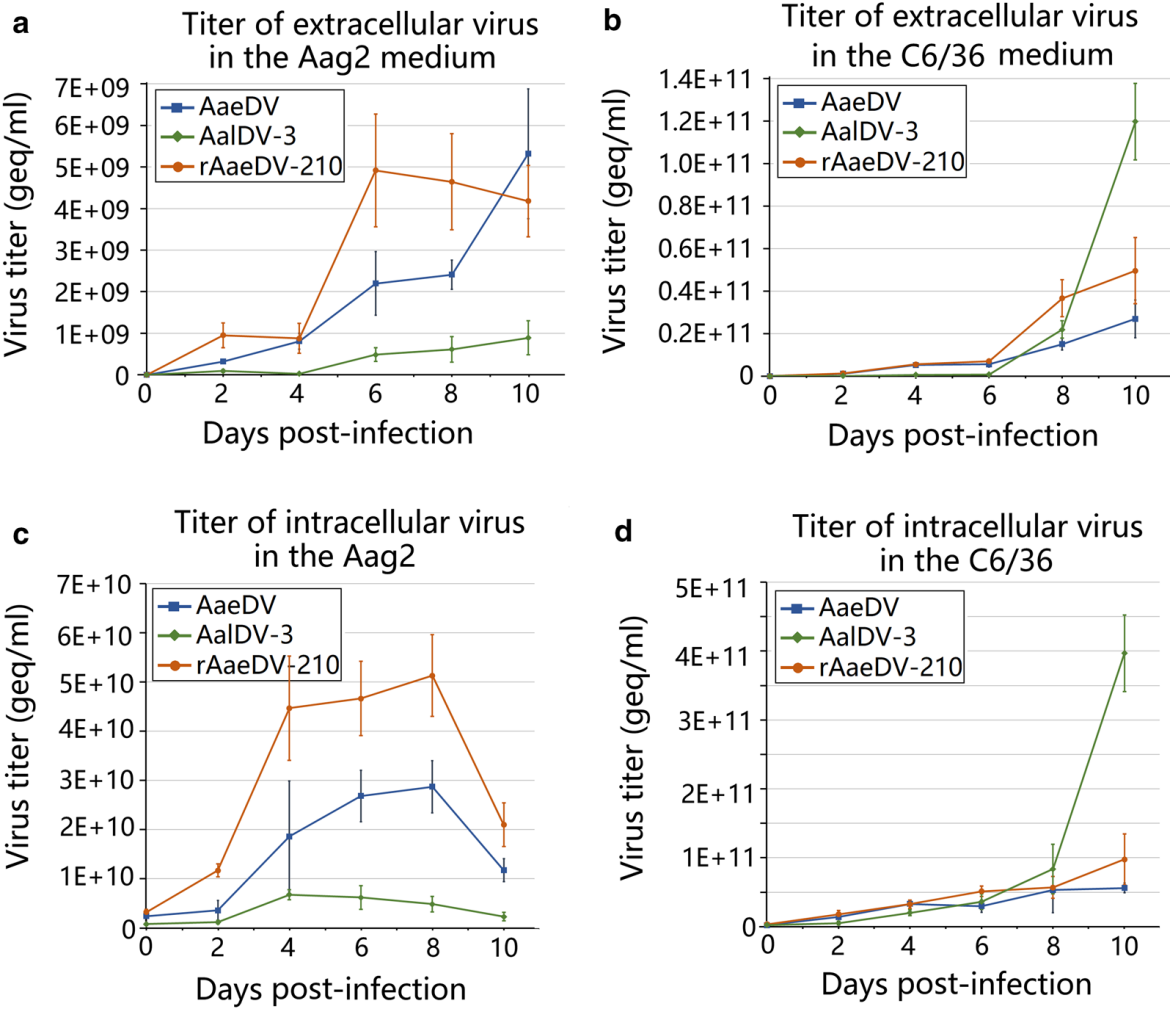
Growth curves of intra- and extracellular MDV titers. The cells were infected with AaeDV, AalDV-3 and recombinant rAaeDV-210. Each time point represents the average titer obtained from three independent experiments with the respective standard deviations. **a** Titer of extracellular virus in the Aag2 cell culture medium. **b** Titer of extracellular virus in the C6/36 cell culture medium. **c** Titer of intracellular virus in the Aag2 cell. **d** Titer of intracellular virus in the C6/36 cell

The proliferation capability of the AaeDV, AalDV-3 and recombinant rAaeDV-210 viruses in Aag2 and C6/36 cells was determined by titer values of both the extracellular viruses from culture and intracellular viruses. The absolute copy number of viral genomic DNA was quantified using SYBR Green I dye in a quantitative PCR assay with MDV-specific primers. The copy numbers of viruses were detected every two days from 0 to 10 dpi (Fig. 1)

In Aag2 cell culture medium, all titers of AaeDV, AalDV-3 and rAaeDV-210 clearly increased from 0 dpi. Both AaeDV and AalDV-3 gradually reached a maximum of 5.32 × 10^9^ ± 1.56 × 10^9^ geq/ml and 8.92 × 10^8^ ± 4.08 × 10^8^ geq/ml, respectively, at 10 dpi, whereas rAaeDV-210 reached a peak (4.92 × 10^9^ ± 1.36 × 10^9^ geq/ml) at 6 dpi and then showed a slight drop. The maximum viral titer of AalDV-3 was significantly lower than that of AaeDV and rAaeDV-210. There was no statistically significant difference in the maximum viral titer between AaeDV and rAaeDV-210 (*t*_(4)_* = *0.26, *P = *0.810). In C6/36 cell culture medium, all titers of AaeDV, AalDV-3 and rAaeDV-210 were rapidly elevated from 0 dpi and reached their maximum values (2.69 × 10^10^ ± 8.84 × 10^9^ geq/ml, 1.20 × 10^11^ ± 1.81 × 10^10^ geq/ml and 4.96 × 10^10^ ± 1.55 × 10^10^ geq/ml, respectively) at 10 dpi. The maximum viral titer of AalDV-3 was higher than that of AaeDV and rAaeDV-210. Moreover, AaeDV showed higher maximum viral titers in the C6/36 cell culture medium compared to that of Aag2 cell culture medium (*t*_(4)_* = *4.13, *P = *0.046).

Intracellular viral titers of AaeDV and rAaeDV-210 in Aag2 cells increased rapidly then reached a peak at 8 dpi (2.87 × 10^10^ ± 5.32 × 10^9^ geq/ml and 5.13 × 10^10^ ± 1.03 × 10^10^ geq/ml, respectively) and subsequently declined; however, AalDV-3 displayed a slight growth trend compared with that of AaeDV and rAaeDV-210, and the maximum titer was observed at 4 dpi (6.78 × 10^9^ ± 1.03 × 10^9^ geq/ml), which then gradually decreased. Intracellular viral titers of AaeDV, AalDV-3 and rAaeDV-210 in C6/36 cells showed a sustainable growth model from 0 dpi to 10 dpi; the highest titers were 5.59 × 10^10^ ± 6.98 × 10^9^ geq/ml, 3.97 × 10^11^ ± 5.54 × 10^10^ geq/ml and 9.77 × 10^10^ ± 3.71 × 10^10^ geq/ml, respectively. The results showed that AaeDV (*t*_(4)_* = *5.351, *P = *0.006) and AalDV-3 (*t*_(4)_* = *12.165, *P = *0.001) showed higher maximum viral titers in the C6/36 cells compared to the Aag2 cells.

### Comparison of viral yields using cells

**Fig. 2:**
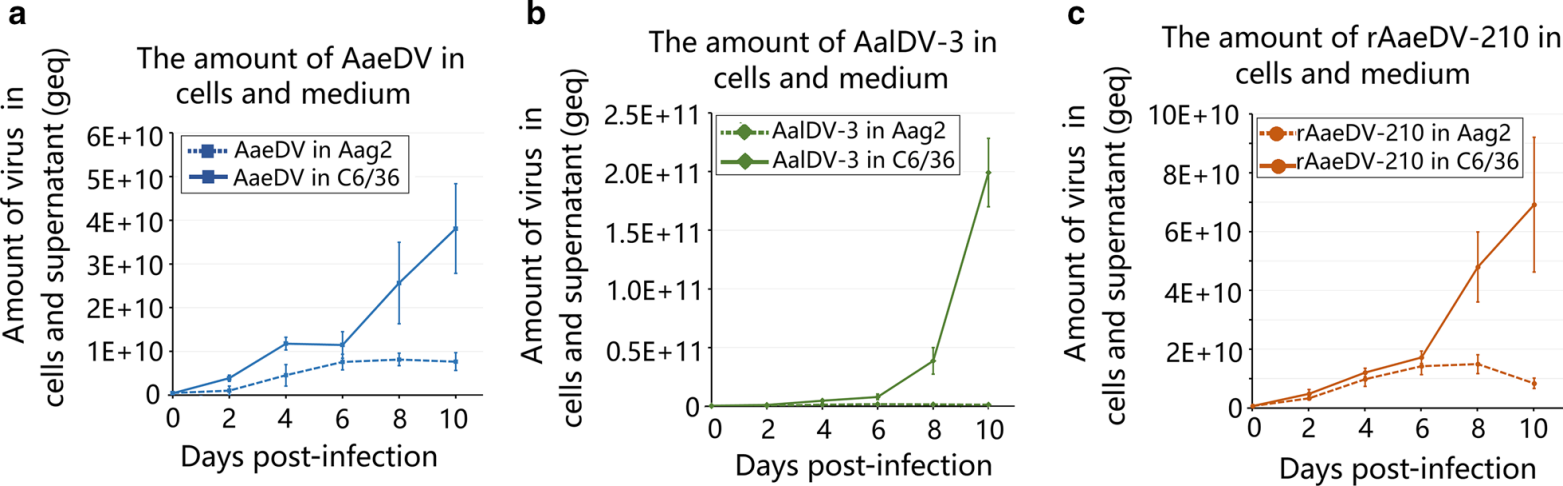
Comparison of the total viral yield in different mosquito cell lines. Total viral yields from 1 ml of medium and 0.2 ml of cells per sample were quantified at serial time points. Each time point represents the average genome copy number obtained from three independent experiments with the respective standard deviations. **a** The amount of AaeDV in cells and medium. **b** The amount of AalDV-3 in cells and medium. **c** The amount of rAaeDV-210 in cells and medium

The total volume of each sample (1 ml of medium and 0.2 ml of cells per sample) and the maximum amount of harvestable virus in the medium and in cells at a certain time points were calculated and compared. As a result, for all three viruses, AaeDV, AalDV-3 and rAaeDV-210, the maximum virus yield was detected in medium (Aag2 medium: 5.32 × 10^9^ ± 1.56 × 10^9^ geq/ml, 8.92 × 10^8^ ± 4.08 × 10^8^ geq/ml and 4.92 × 10^9^ ± 1.36 × 10^9^ geq/ml, respectively; C6/36 medium: 2.69 × 10^10^ ± 8.84 × 10^9^ geq/ml, 1.20 × 10^11^ ± 1.81 × 10^10^ geq/ml and 4.96 × 10^10^ ± 1.55  × 10^10^ geq/ml respectively) and not in cells (Aag2: 2.35 × 10^9^ ± 4.65 × 10^8^ geq/ml, 1.36 × 10^9^ ± 2.06 × 10^8^ geq/ml and 1.03 × 10^10^ ± 2.07 × 10^9^ geq/ml, respectively; C6/36: 1.12 × 10^10^ ± 1.40 × 10^9^ geq/ml, 7.94 × 10^10^ ± 1.11 × 10^10^ geq/ml and, 1.95 × 10^10^ ± 7.41 × 10^9^ geq/ml respectively) (Fig. 2a–c), which indicated that a large amount of densoviruses were persistently released from infected mosquito cells and continuously accumulated in medium.

**Fig. 3:**
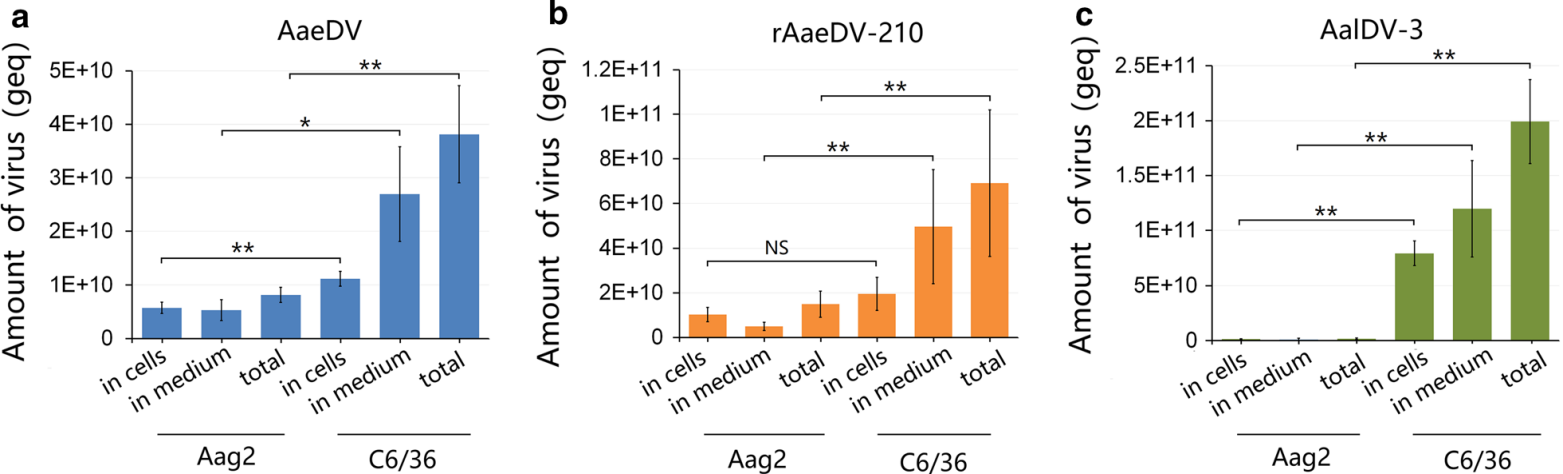
Comparison of the maximum viral yield obtained using Aag2 and C6/36 cells. The maximum viral yield obtained from cells, medium and the sum of intra- and extracellular viruses were quantified. Data are representative of three independent experiments, and values are expressed as the mean ± SD; bars represent the standard error (*n * =  3). *P < 0.05, **P < 0.01, NS, no significant difference. **a** AaeDV. **b** rAaeDV-210. **c** AalDV-3

Moreover, the yield sums of three kinds of viruses in cells and medium were quantified. For virus production, viral yield reached a peak at 6 or 8 dpi using Aag2 cells, whereas viral yield gradually increased over a prolonged time in C6/36 cells. The total yield of AaeDV, AalDV-3 and rAaeDV-210 in C6/36 cells was nearly 5, 115 and 5 times higher than that of Aag2, respectively. In addition, the maximum yield of recombinant rAaeDV-210 virus did not exhibit a significant difference compared with its original wild-type AaeDV (Fig. 3) both in C6/36 cells (*t*_(4)_* = *1.58, *P = *0.189) and Aag2 cells (*t*_(4)_* = *1.95, *P = *0.124).

### Proliferation capability of MDVs in larvae

The proliferation capability of the AaeDV, AalDV-3 and recombinant rAaeDV-210 viruses in *Ae. albopictus*, *Ae. aegypti* and *Cx. quinquefasciatus* larvae was determined by absolute quantification. The quantification method detected the copy numbers of viruses every two days from 1 to 11 dpi in 1st–2nd-instar larvae and from 1 to 7 dpi in 3rd–4th-instar larvae.

**Fig. 4:**
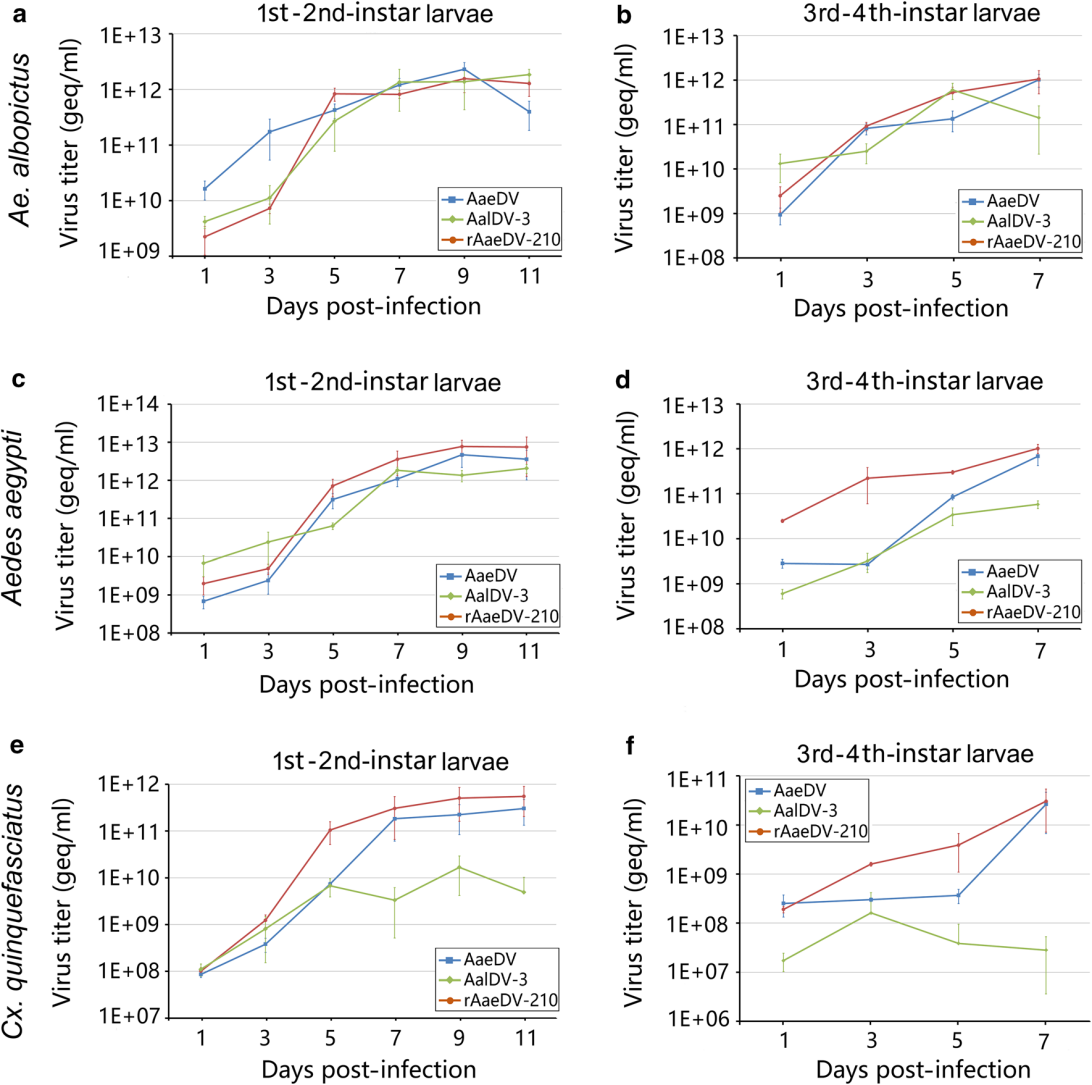
The accumulation of MDV in mosquito larvae. The 1st–2nd-instar and 3rd–4th-instar larvae of *Ae. albopictus*, *Ae. aegypti* and *Cx. quinquefasciatus* (*n = *100 per group) were infected with AaeDV, AalDV-3 and recombinant rAaeDV-210. Each time point represents the average titer obtained from three independent experiments with the respective standard deviations. Viral titers in *Ae. albopictus* 1st–2nd-instar larvae (**a**) and 3rd–4th-instar larvae (**b**); *Ae. aegypti* 1st–2nd-instar larvae (**c**) and 3rd–4th-instar larvae (**d**); and *Cx. quinquefasciatus* 1st–2nd-instar larvae (**e**) and 3rd–4th-instar larvae (**f**)

In 1st–2nd-instar larvae of *Ae. albopictus*, the titers of AaeDV, AalDV-3 and rAaeDV-210 showed exponential growth from 1 dpi. Both AaeDV and rAaeDV-210 reached their maximal titers (2.30 × 10^12^ ± 7.29 × 10^11^ geq/ml and 1.56 × 10^12^ ± 6.86 × 10^11^ geq/ml, respectively) at 9 dpi and then decreased slightly at 11 dpi, whereas the AalDV-3 titer reached its highest level (1.82 × 10^12^ ± 4.4 × 10^11^ geq/ml) at 11 dpi (Fig. 4a). In 3rd–4th-instar larvae of *Ae. albopictus*, all the viral titers also displayed an obvious growth trend with time, and the maximum viral titer of AaeDV and rAaeDV-210 (1.02 × 10^12^ ± 3.24 × 10^11^ geq/ml and 1.05 × 10^12^ ± 5.62 × 10^11^ geq/ml, respectively) were detected at 7 dpi. The maximum titer of AalDV-3 (6.01 × 10^11^ ± 2.36 × 10^11^ geq/ml) was detected at 5 dpi (Fig. 4b).

The proliferation of viruses in 1st–2nd-instar and 3rd–4th-instar larvae of *Ae. aegypti* displayed similar characteristics to that in *Ae. albopictus.* Both AaeDV and rAaeDV-210 reached their maximum values in 1st–2nd-instar larvae at 9 dpi (4.67 × 10^12^ ± 2.49 10^12^ geq/ml and 7.70 × 10^12^ ± 3.48 × 10^12^ geq/ml, respectively) and AalDV-3 (2.06 × 10^12^ ± 5.38 × 10^11^ geq/ml) at 11 dpi (Fig. 4c). In 3rd–4th-instar larvae of *Ae. aegypti*, the titers of AaeDV, AalDV-3 and rAaeDV-210 reached their maximum values (6.83 × 10^11^ ± 2.56 × 10^11^ geq/ml, 5.80 × 10^10^ ± 1.12 × 10^10^ geq/ml and 1.02 × 10^12^ ± 2.37 × 10^11^ geq/ml, respectively) at 7 dpi (Fig. 4d).

The growth trends of viruses in *Cx. quinquefasciatus* larvae were almost identical to those in *Aedes*. The maximum concentrations of AaeDV and rAaeDV-210 were 3.03 × 10^11^ ± 1.71 × 10^11^ geq/ml and 5.50 × 10^11^ ± 3.48 × 10^11^ geq/ml, respectively, at 11 dpi in 1st–2nd-instar larvae; however, AalDV-3 at 9 dpi showed a distinctive trend, decreasing by more than one order of magnitude (1.68 × 10^10^ ± 1.26 × 10^10^ geq/ml) (Fig. 4e). In 3rd–4th-instar larvae at 9 dpi, all of the vial titers of AaeDV, AalDV-3 and rAaeDV-210 reached their highest levels (2.64 × 10^10^ ± 1.97 × 10^10^ geq/ml, 1.61 × 10^8^ ± 2.55 × 10^8^ and 3.03 × 10^10^ ± 2.32 × 10^10^ geq/ml, respectively) at 7 dpi (Fig. 4f).

### The trend of viral titers in infected larval rearing water

**Fig. 5:**
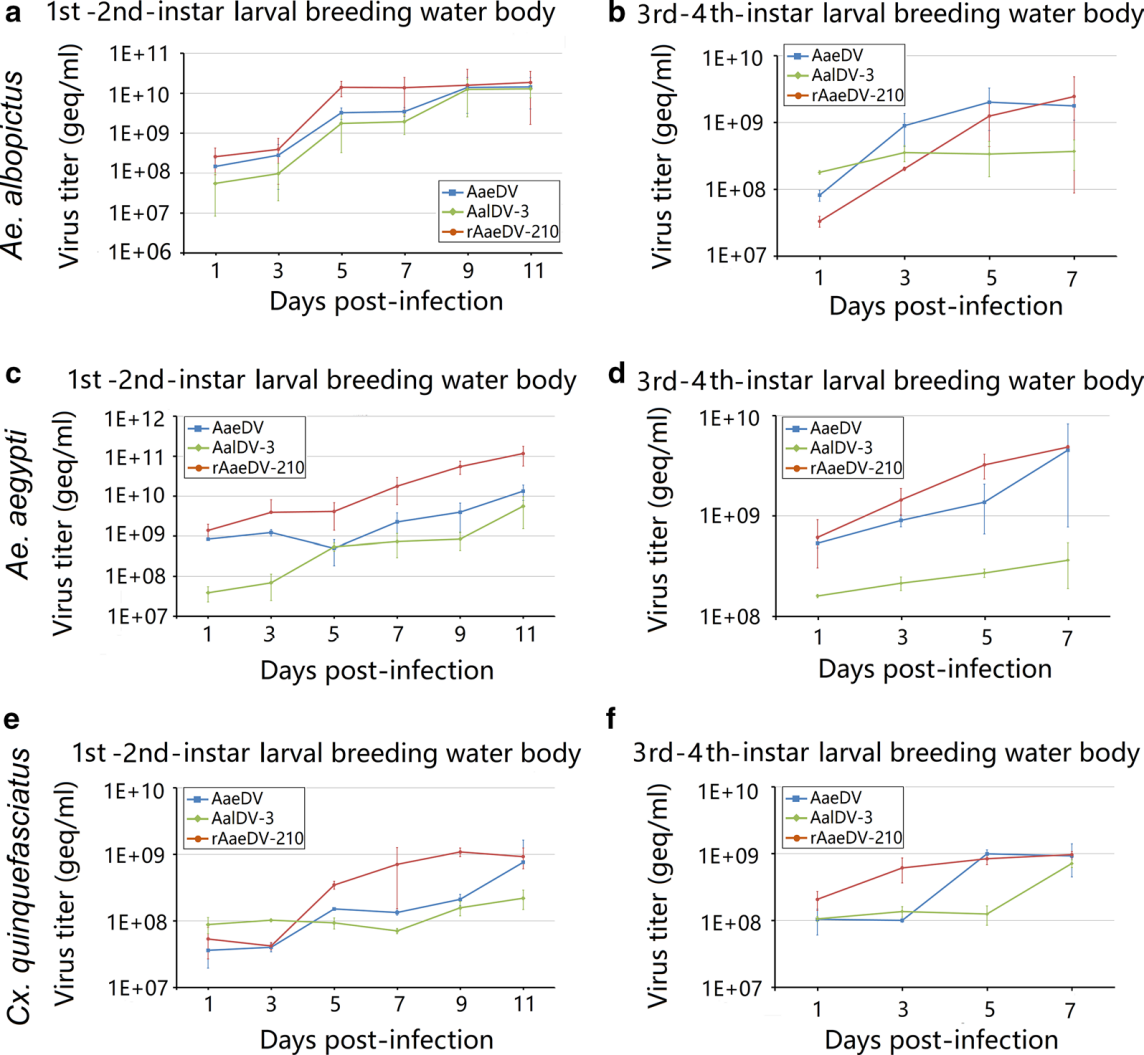
The accumulation of MDV in larval rearing water. The viral yields from larval rearing water were quantified at serial time points. Each time point represents the average titer obtained from three independent experiments with the respective standard deviations. The titer of AaeDV, AalDV-3 and recombinant rAaeDV-210 in *Ae. albopictus* 1st–2nd-instar larval rearing water (**a**) and 3rd–4th-instar larval rearing water (**b**); *Ae. aegypti* 1st–2nd-instar larval rearing water (**c**) and 3rd–4th-instar larval rearing water (**d**); and *Cx. quinquefasciatus* 1st–2nd-instar larval rearing water (**e**) and 3rd–4th-instar larval rearing water (**f**)

The viral titers in the rearing water of three species of larvae were also quantified, and the samples were collected at the same time points as described for larvae. The result is exciting; the virus infection was prolonged, and all three viruses showed a similar consecutive growth pattern in both 1st–2nd-instar and 3rd–4th-instar larvae to that of all three species *in vivo*. Although the highest concentration of virus in the water was slightly lower than that *in vivo*, viral titers still presented the highest values at 11 dpi in rearing water of *Ae. albopictus* 1st–2nd-instar larvae (Fig. 5a), at 5 dpi (AaeDV) and 7 dpi (AalDV-3 and rAaeDV-210) in rearing water of 3rd–4th-instar larvae (Fig. 5b); at 11 dpi in rearing water of *Ae. aegypti* 1st–2nd-instar larvae (Fig. 5c), at 7 dpi in rearing water of 3rd–4th-instar larvae (Fig. 5d); at 11 dpi (AaeDV and AalDV-3) and 9 dpi (rAaeDV-210) in *Cx. quinquefasciatus* 1st–2nd-instar larvae (Fig. 5e), at 5 dpi (AaeDV) and 7 dpi (AalDV-3 and rAaeDV-210) in rearing water of 3rd–4th-instar larvae (Fig. 5f).

### Comparison of the viral yield with the *in vivo* production method

**Fig. 6:**
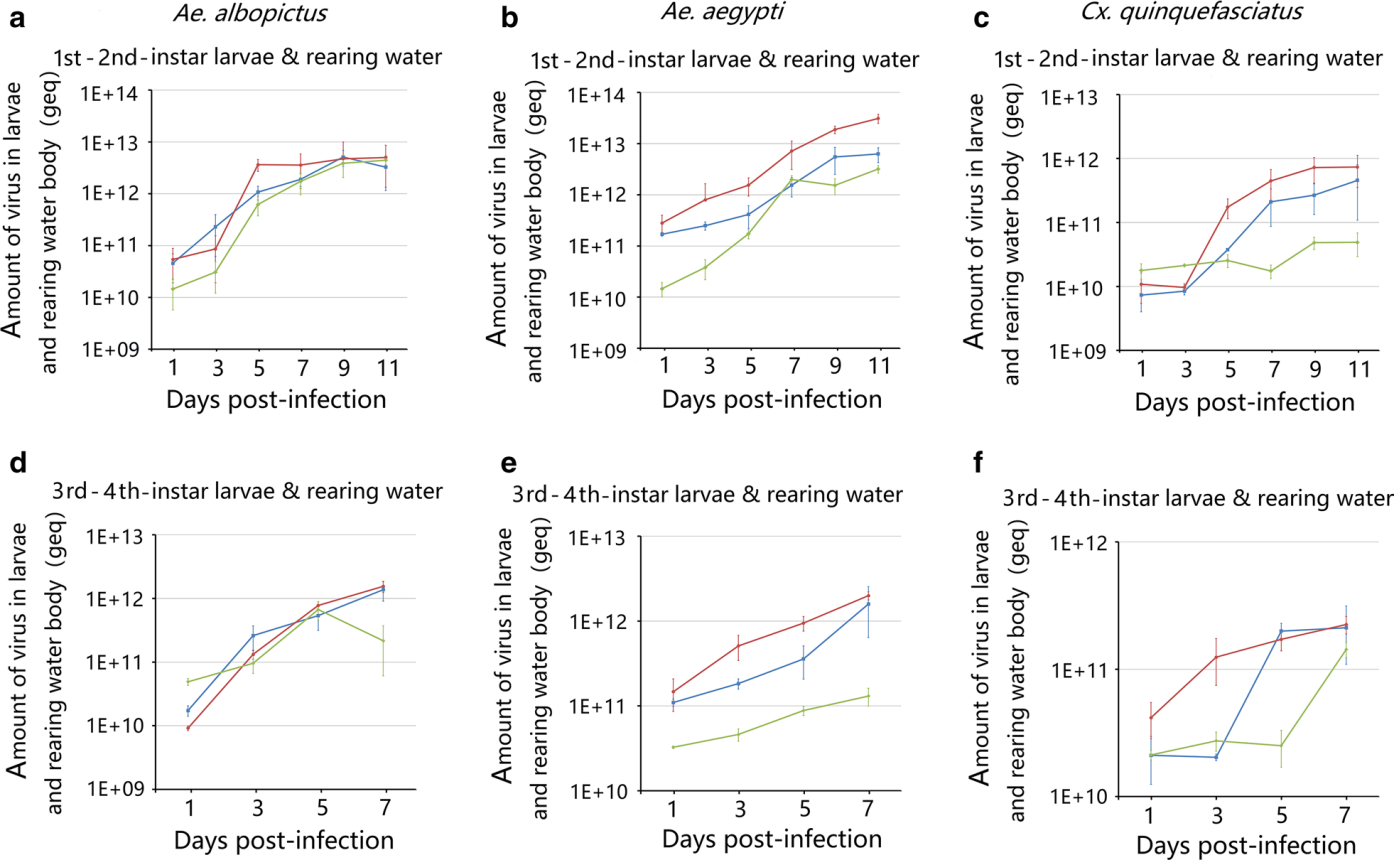
Comparison of total viral yield over time in different larvae systems. Total viral yields from 200 ml of larvae rearing water and 1 ml of cells per sample were quantified at serial time points. Each time point represents the average genome copy number obtained from three independent experiments with the respective standard deviations. The amount of AaeDV, AalDV-3 and rAaeDV-210 in *Ae. albopictus* 1st–2nd-instar larvae and larval rearing water (**a**); *Ae. aegypti* 1st–2nd-instar larvae and larval rearing water (**b**); *Cx. quinquefasciatus* 1st–2nd-instar larvae and larval rearing water (**c**); *Ae. albopictus* 3rd–4th-instar larvae and larval rearing water (**d**); *Ae. aegypti* 3rd–4th-instar larvae and larval rearing water (**e**); *Cx. quinquefasciatus* 3rd–4th-instar larvae and larval rearing water (**f**)

Regarding overall viral yields, the total amount of obtainable virus in larvae and larval rearing water were calculated by multiplying the sample volume (1 ml of larvae and 200 ml of water, respectively) and viral concentration. To evaluate the trend of virus yield growth, the total virus yield at different time points was calculated. The total viral product showed a notable growth trend in infected *Ae. albopictus* 1st–2nd-instar larvae where the amount of AaeDV, AalDV-3 and rAaeDV-210 reached a peak (5.10 × 10^12^ ± 1.74  × 10^12^ geq, 4.44 × 10^12^ ± 7.85 × 10^11^ geq and 5.01 × 10^12^ ± 3.67 × 10^12^ geq, respectively) at 9, 11 and 11 dpi, respectively (Fig. 6a). In both *Ae. aegypti* and *Cx. quinquefasciatus*, infected 1st–2nd-instar larvae, the AaeDV, AalDV-3 and rAaeDV-210 amounts reached a maximum at 11 dpi (*Ae. aegypti:* 6.27 × 10^12^ ± 2.05 × 10^12^ geq, 3.19 × 10^12^ ± 5.39 × 10^11^ geq and 3.09 × 10^13^ ± 6.31 × 10^12^ geq, respectively; *Cx. quinquefasciatus*: 4.56 × 10^11^ ± 3.47 × 10^11^ geq, 4.89 × 10^10^ ± 1.96 × 10^10^ geq and 7.36 × 10^11^ ± 3.83  × 10^11^ geq, respectively) (Fig. 6b, c). Compared with *Ae. albopictus* 1st–2nd-instar larvae, viral yield showed a relatively slight increase when using 3rd–4th-instar larvae to produce virus. AaeDV, AalDV-3 and rAaeDV-210 reached a maximum (1.37 × 10^12^ ± 4.64 × 10^11^ geq, 6.69 × 10^11^ ± 2.10 × 10^11^ geq and 1.55 × 10^12^ ± 3.05 × 10^11^ geq, respectively) at 7, 5 and 7 dpi, respectively (Fig. 6d). In both *Ae. aegypti* and *Cx. quinquefasciatus* infected 3rd–4th-instar larvae, the AaeDV, AalDV-3 and rAaeDV-210 amounts reached a maximum at 11 dpi (*Ae. aegypti:* 1.59 × 10^12^ ± 9.50 × 10^11^ geq, 1.31 × 10^11^ ± 3.12 × 10^10^ geq and 1.99 × 10^12^ ± 2.14 × 10^11^ geq, respectively; *Cx. quinquefasciatus*: 2.12 × 10^11^ ± 1.03 × 10^11^ geq, 1.43 × 10^11^ ± 1.93 × 10^10^ geq and 2.24 × 10^11^ ± 3.52 × geq, respectively) (Fig. 6e, f).

**Fig. 7:**
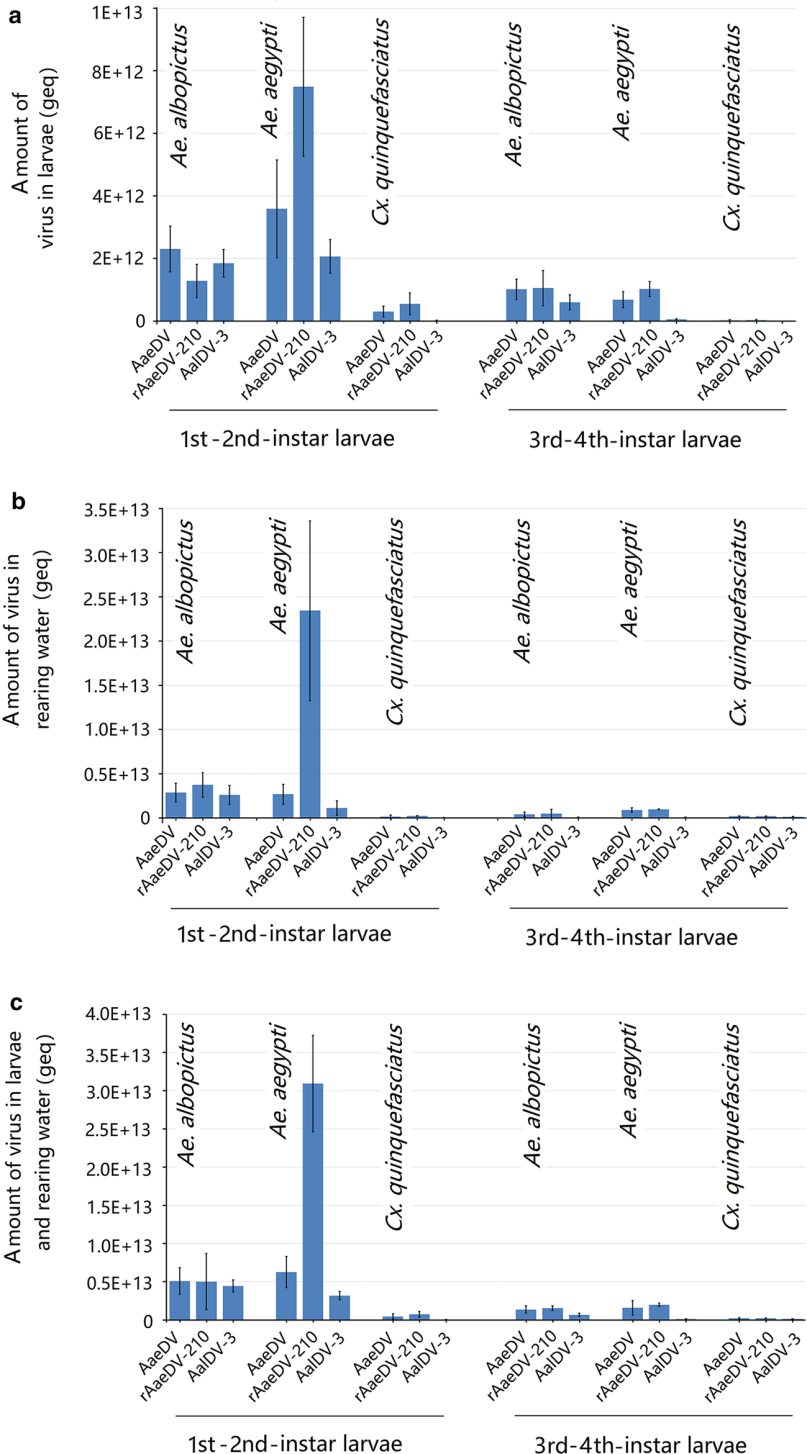
Comparison of maximum viral yield in different larvae systems. The maximum viral yields obtained from larvae, rearing water and the sum of the combination were quantified. Data are representative of three independent experiments, and values are expressed as the mean ± SD. **a** The maximum viral yield obtained from larvae. **b** The maximum viral yield obtained from larval rearing water. **c** The maximum viral yield sum of the combination

Regarding the total product, the maximal viral yields in the 1st–2nd-instar and 3rd–4th-instar larvae of all three species of mosquitoes were compared. The results showed that for all viruses, significantly higher viral yields were obtained using 1st–2nd-instar larvae rather than 3rd–4th-instar larvae, regardless of whether *in vivo* (Fig. 7a), in the water (Fig. 7b) or the combined total (Fig. 7c). Moreover, when comparing the 1st–2nd-instar larvae of the three mosquito species, higher yields were detected in *Aedes* mosquitoes than in *Culex* mosquitoes *in vivo*, in the water body or combination. However, we did not detect significant differences between *Ae. aegypti* and *Ae. albopictus*, except for the rAaeDV-210, which showed higher yields using *Ae. aegypti* 1st–2nd-instar larvae.

### Comparison of the infection percentage of different mosquito larvae

**Fig. 8:**
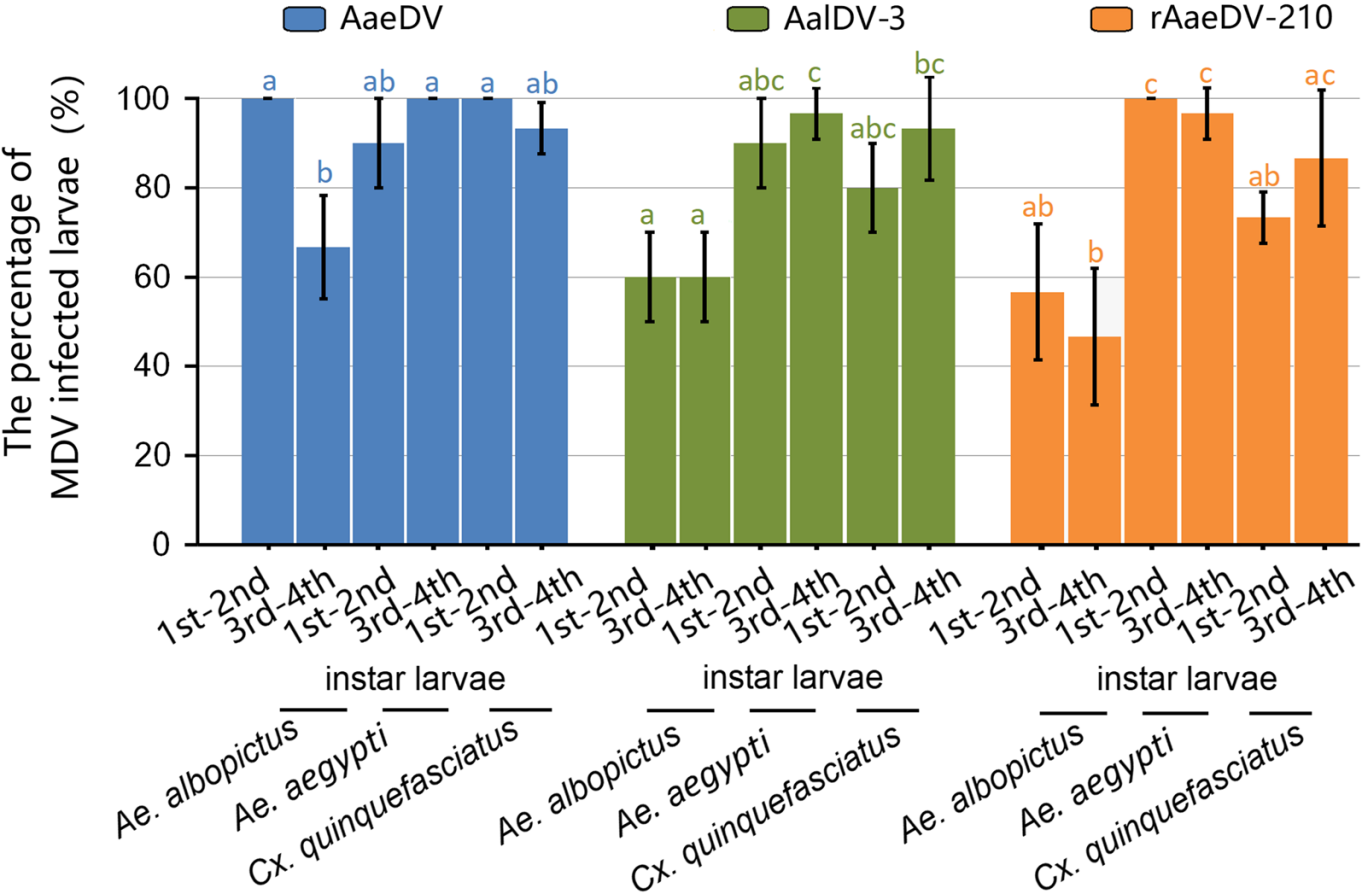
The percentage of MDV infected larvae. Viral DNA was detected by the traditional PCR method with gene-specific primers for the genomic conserved region. The percentage of MDV infected larvae was determined using AaeDV, AalDV-3 and rAaeDV-210 to infect the 1st–2nd-instar and 3rd–4th-instar larvae of *Ae. albopictus*, *Ae. aegypti* and *Cx. quinquefasciatus.* The data are representative of three independent experiments, and the values are expressed as the mean ± SD. Different letters with the same color above bars represent significant differences in relative expression levels at the *P *≤ 0.05 level

To explore the infection percentage of mosquito larvae that become infected when exposed to MDV, the genomic PCR method was used to analyze the infection percentages of AaeDV, AalDV-3 and rAaeDV-210 in *Ae. albopictus*, *Ae. aegypti* and *Cx. quinquefasciatus* larvae at 24 hours post-infection. The infection rates in 1st–2nd-instar and 3rd–4th-instar larvae were also compared to confirm the relationship between developmental stage and infection rate. For the 1st–2nd-instar larvae, *Ae. aegypti* showed greater susceptibility to rAaeDV-210 than *Ae. albopictus* (*χ*^2^* = *16.60, *df = *1, *P* < 0.0001), and greater susceptibility to rAaeDV-210 than *Cx. quinquefasciatus* (*χ*^2^* = *10.22, *df = *1, *P = *0.001) (Fig. 8). For the 3rd–4th-instar larvae, *Ae. aegypti* showed a greater susceptibility to AaeDV (*χ*^2^* = *12.00, *df = *1, *P* < 0.001) and rAaeDV-210 (*χ*^2^* = *21.82, *df = *1, *P* < 0.0001) than *Ae. albopictus*, whereas, no significant differences were observed between *Ae. aegypti* and *Cx. quinquefasciatus* (AaeDV: *χ*^2^* = *2.07, *df = *1, *P* =  0.150; AalDV-3: *χ*^2^* = *0.35, *df = *1, *P* =  0.554). These results indicate that the different infection percentages in mosquito species were not an important factor affecting the viral yield, and it seems that *Aedes* mosquito larvae were more suitable for viral proliferation and release than *Culex* mosquito larvae.

## Discussion

Chemical pesticides have been widely used over the past 60 years in agricultural pest control and in response to epidemics [3, 19]. Today, over 2.3 billion kilograms of pesticides are used annually worldwide [20]. However, the excessive use of chemical pesticides is accompanied by a growing general concern for the negative impacts on the ecosystem and human health. Globally, chemical insecticides directly contribute to more than 100,000 deaths and two million hospital admissions every year [21]. Additionally, intensive prophylactic application causes the pests to rapidly develop resistance to most traditional insecticides and causes widespread concern over chemical pesticide abuse [22–24], resulting in the imperative requirement of alternative and ecologically friendly biocontrol approaches.

Entomopathogenic viruses have a narrow host spectrum, are non-toxic to humans and non-target organisms, have a long shelf life and are eco-friendly; these favorable properties make entomopathogenic viruses a potentially great bioinsecticide. Currently, more than 240 insect virus isolates have been isolated from 196 species from 35 families and seven orders in China. Since the first viral insecticide, *Helicoverpa armigera* nucleopolyhedrovirus (HaNPV), was registered in 1993 [25], a total of 69 products have been authorized as commercial pesticides by the Ministry of Agriculture of China (MAC) through June 2018 [26]. In the last five years, it has been estimated that 1600 metric tons of viral insecticidal formulations were produced annually, representing nearly 0.2% of the total pesticide yield in China [27].

With the widespread use of viral insecticides in agriculture, the process of propagating viruses for vector control is tortuous, especially in mosquitoes. At present, the major entomopathogenic viruses that display pathogenicity to mosquitoes are NPVs, CPVs, MDVs and MIVs. Although baculovirus application has been successful for agricultural insects, the host specificity of CuniNPV is confined to species of the mosquito genus *Culex* [7]. Moreover, the virus’ slow speed of action limits its application in mosquitoes [28]. MIVs showed only moderate infection in mosquito larvae, and mortality rates were typically lower than 25% in first- and second-instar larvae [29]. Although relatively higher rates were observed in CPV-infected larvae, the weak pathogenicity was the major obstacle for their development and application. For example, even heavily infected larvae could often survive to pupation and emergence [29]. As a result, the only prospect for potential commercial production is MDV. MDVs have displayed several promising characteristics, such as a higher infection rates for all developmental larval stages, especially molting larvae. Furthermore, newly proliferated virus can be sustained and released into water from the infected and dead mosquito larvae, thereby persisting or increasing the viral titers and allowing for horizontal transmission to uninfected or newly hatched larvae. Additionally, MDVs can be transmitted vertically to their offspring by infected females [30–32]. We previously reported the development of a non-defective recombinant AaeDV, which can express small interference RNAs using an intronic sRNA expression strategy that lays the foundation for further enhancing the virulence of MDVs for bioinsecticide use [12, 18]. AaeDV as a product (Viroden) was evaluated in small-scale field studies in the former Soviet Union in 1979 [30]. However, to date, substantial progress has not been made. The major obstacle was that viruses must be produced in mosquito cell lines, so the production process was therefore both expensive and time-consuming. A suspension culture of C6/36 cells in serum-free protein-free Sf-900 II media was previously developed by Suchman et al. [33], and a higher density of cells was collected than that from the conventional process. However, the maximum AaeDV titer only reached 3.8 × 10^8^ geq/ml for AeDNV contained in 45 ml of culture medium. Moreover, the price of commercial Sf-900 II was too high to mass produce for large-scale field applications. Considering the effect of MDVs on the mosquito larvae in a concentration-dependent manner, applied viral titers should also be taken into account [34–36].

In our study, two wt MDVs, AaeDV and AalDV-3, and a recombinant rAalDV-210 were used as “seeds”; two mosquito cell lines, Aag2 and C6/36, and the larvae of three species, *Ae. albopictus*, *Ae. aegypti* and *Cx. quinquefasciatus*, were used as the viral production “factories”, and the viral yield was compared. Two criteria, virus titer and viral yield, were used to evaluate the production capacity of these “factories”. Considering that a feature of MDVs is that they can sustain release into the culture medium and rearing water from the infected cells, we also counted the two criteria *in vivo*, *in vitro* and combined. Our results showed that (i) the three viruses displayed higher maximum virus titers in cells and larvae than in culture medium and rearing water; (ii) the three viruses displayed higher maximum viral yields in medium and water; (iii) the three viruses displayed higher total maximum viral yields in C6/36 cells than in Aag2 cells; (iv) the three viruses displayed higher total maximum viral yields in *Aedes* mosquitoes than in *Culex* mosquitoes; (v) higher viral yields were produced by infection of 1st–2nd-instar larvae compared to 3rd–4th-instar larvae; and (vi) the recombinant virus did not display significantly lower yields than that of the wt viruses in nearly all the samples. In summary, by using 100 1st–2nd-instar *Aedes* mosquito larvae in 200 ml of rearing water, more than 10^13^ geq MDV yield can be obtained. Considering the lower breeding cost (0.01 US$ per 5 g of turtle food pellet), this *in vivo* method shows great potential for the large-scale production of MDVs.

## Conclusions

In this study, the three viruses we selected display similar infection rates in *Ae. albopictus*, *Ae. aegypti* and *Cx. quinquefasciatus*, and the lower viral yield may be a result of the higher death rate of MDVs in *Cx. quinquefasciatus.* As previously described, infectivity ratios may vary for different viruses and different mosquito species [11, 36, 37]. It is still essential to test different species of mosquito larvae and to evaluate the most suitable candidates before beginning large-scale production. As a successful commercial reference, PfDNV (trade name Biokiller) was the first authorized commercial densovirus insecticide in China in 2008, with an annual output of 2000 tons in recent years [15]. MDVs exhibit promising prospects and great potential for mosquito control in the future.

## Additional file

**Additional file 1: Table S1.** Primers and amplification conditions for PCR and qPCR assays.

## Data Availability

The datasets supporting the conclusions of this article are included within the article and its additional file.

## Abbreviations

MBDs: mosquito-borne diseases
MDVs: mosquito densoviruses
AaeDV: *Ae. aegypti* densoviruses
AalDV-3: *Ae. albopictus* densoviruses-3
rAaeDV-210: recombinant *Ae. aegypti* densoviruses-210
geq: genome equivalents/ml
IMM: integrated mosquito management
IVs: inclusion viruses
NIVs: non-inclusion viruses
NPVs: nuclear polyhedrosis viruses
CuniNPV: *Culex nigripalpus* nuclear polyhedrosis virus
CPVs: cytoplasmic polyhedrosis viruses
MIVs: mosquito iridoviruses
DVs: densoviruses
ssDNA: single-stranded DNA
shRNA: short hairpin RNA
miRNA: microRNA
PfDNV: *Periplaneta fuliginosa* densonucleosis virus
qPCR: quantitative real-time PCR
HaNPV: *Helicoverpa armigera* nucleopolyhedrovirus
MAC: Ministry of Agriculture of China
SD: standard deviation
